# Impact of State‐Level Equity Indices on Psychosocial Outcomes Among Sexual and Gender Minority Caregivers of People with Alzheimer’s Disease and Related Dementias

**DOI:** 10.1002/alz.089339

**Published:** 2025-01-09

**Authors:** Joel G Anderson, Krystal R. Kittle, Joseph Winberry, Kari A. Hancock, Jordan Westcott, Namrata Mukherjee, Jason D. Flatt

**Affiliations:** ^1^ College of Nursing, University of Tennessee, Knoxville, TN USA; ^2^ University of Massachusetts‐Amherst, Amherst, MA USA; ^3^ University of North Carolina‐Chapel Hill, Chapel Hill, NC USA; ^4^ University of Tennessee‐Knoxville, Knoxville, TN USA; ^5^ University of Tennessee, Knoxville, TN USA; ^6^ University of Nevada Las Vegas School of Public Health, Las Vegas, NV USA

## Abstract

**Background:**

Our previous work has found that sexual and gender minority (SGM) or LGBTQIA+ caregivers of people living with Alzheimer’s disease and related dementias (ADRD) experience higher levels of stigma, depressive symptoms, and stress than non‐SGM caregivers and that these outcomes are associated with experiences of microaggressions related to their SGM identities. Guided by the Health Equity Promotion Model, we sought to explore the impact of the environmental context on psychosocial outcomes among SGM caregivers of people living with ADRD.

**Methods:**

Data from a cross‐sectional online survey using a non‐probabilistic sample of SGM caregivers of people with ADRD recruited via social media (*n* = 284) were combined with publicly available data reporting composite equity climate index scores across five domains: legal/non‐discrimination protections, youth/family support, political/religious attitudes, health access/safety, and work environment/employment. The bimodal distribution of states by total equity climate score was used to create a low/high‐score variable for comparisons in global health status, lifetime and day‐to‐day discrimination, lifetime victimization, microaggression, perceived stress, family quality of life, caregiver stigma, and depressive symptoms.

**Results:**

SGM caregivers of people with ADRD in states with lower equity climate scores reported significantly more experiences of microaggressions (*p* = 0.036), lower levels of perceived stress (*p* = 0.050) and family quality of life (*p* = 0.037), and higher levels of caregiver stigma (*p*<0.001) and depressive symptoms (*p*<0.001) compared with SGM caregivers living in states with higher equity climate scores. Gay caregivers living in states with lower equity climate scores reported levels of caregiver stigma 18% higher than those living in states with higher equity scores (*p* = 0.004). There were no significant differences in global health status, lifetime and day‐to‐day discrimination, and lifetime victimization by equity climate scores.

**Conclusion:**

The environmental context of SGM lived experiences influences psychosocial outcomes related to ADRD caregiving. These differences may be driven by the unique minority stressors experienced by SGM caregivers of people with ADRD, which are known to influence the overall physical and mental health of the SGM population. Further research is warranted to understand the impact of these environmental influences on SGM caregivers of people with ADRD to develop supports and policy approaches to promote well‐being.

